# The newly proposed Metabolic Score for Visceral Fat is a reliable tool for identifying non-alcoholic fatty liver disease, requiring attention to age-specific effects in both sexes

**DOI:** 10.3389/fendo.2023.1281524

**Published:** 2023-11-27

**Authors:** Maobin Kuang, Jiajun Qiu, Dongdong Li, Chong Hu, Shuhua Zhang, Guotai Sheng, Yang Zou

**Affiliations:** ^1^ Department of Internal Medicine, Medical College of Nanchang University, Jiangxi Provincial People’s Hospital, Nanchang, Jiangxi, China; ^2^ Jiangxi Cardiovascular Research Institute, Jiangxi Provincial People’s Hospital, The First Affiliated Hospital of Nanchang Medical College, Nanchang, Jiangxi, China; ^3^ Jiangxi Provincial Geriatric Hospital, Jiangxi Provincial People’s Hospital, The First Affiliated Hospital of Nanchang Medical College, Nanchang, Jiangxi, China; ^4^ Department of Pulmonary and Critical Care Medicine, Jiangxi Provincial People’s Hospital, The First Affiliated Hospital of Nanchang Medical College, Nanchang, Jiangxi, China; ^5^ Department of Gastroenterology, Jiangxi Provincial People’s Hospital, The First Affiliated Hospital of Nanchang Medical College, Nanchang, Jiangxi, China

**Keywords:** NAFLD, Metabolic Visceral Fat Score, sex, METS-VF, visceral adipose tissue

## Abstract

**Objective:**

The newly proposed Metabolic Visceral Fat Score (METS-VF) is considered a more effective measure for visceral adipose tissue (VAT) than other obesity indicators. This study aimed to reveal the association between METS-VF and non-alcoholic fatty liver disease (NAFLD), and its variations across age groups within both sexes.

**Methods:**

Data from 14,251 medical examiners in the NAGALA project were employed in this study. 3D fitted surface plots were constructed based on multivariate logistic regression models to visualize the isolated and combined effects of aging and METS-VF on NAFLD. Receiver operating characteristic curve (ROC) analysis was conducted to compare the diagnostic performance of METS-VF with other VAT surrogate markers in predicting NAFLD.

**Results:**

The results of multivariate logistic regression analysis showed that each unit increase in METS-VF was independently associated with a 333% and 312% increase in the odds of NAFLD in males and females, respectively. Additionally, the 3D fitted surface plot showed that age significantly influenced the association between METS-VF and the odds of NAFLD in both sexes, as follows: (i) In males, when METS-VF was less than 6.2, the METS-VF-related odds of NAFLD increased gradually with age in the 20-45 age group, reached a plateau in the 45-65 age group, and then decreased in the group above 65 years old; however, when male METS-VF exceeded 6.2, aging and METS-VF combined to further increase the odds of NAFLD in all age groups, particularly in the 45-65 age group. (ii) In females, aging seemed to reduce METS-VF-related odds of NAFLD in the 18-40 age group, but significantly increased it in the 40-60 age group, particularly for those with higher METS-VF levels. Further ROC analysis revealed that compared to other VAT surrogate markers, METS-VF showed the highest diagnostic accuracy for NAFLD in females, especially in those under 45 years of age [area under the curve (AUC) = 0.9256].

**Conclusions:**

This study firstly revealed a significant positive correlation between METS-VF and the odds of NAFLD, with METS-VF surpassing other VAT surrogate markers in NAFLD diagnosis. Moreover, age significantly influenced the METS-VF-related odds of NAFLD and METS-VF’s diagnostic efficacy for NAFLD in both sexes.

## Introduction

NAFLD is currently the most common metabolic liver disease, with a global prevalence of approximately 25%, it is the main cause of cardiometabolic diseases and severe liver diseases (1, 2). Within the field of metabolic diseases, the continued reliance on liver tissue biopsy for the diagnosis of NAFLD and the lack of approved treatments for NAFLD to date are two of the most important challenges that exist today (3). Therefore, primary prevention based on the major risk factors of NAFLD and further exploration of diagnostic or prognostic biomarkers are crucial in mitigating the global trend of NAFLD prevalence (4).

It is well known that obesity is one of the most important modifiable risk factors for NAFLD, and in particular, the level of VAT is closely related to the occurrence and development of NAFLD (5, 6). VAT is the white adipose tissue surrounding the internal organs in the human abdomen, which plays an important role in protecting internal organs, providing energy reserves, regulating metabolic balance and affecting the functions of the internal organs (7, 8). However, excessive VAT deposition around metabolic organs such as liver and pancreas will lead to metabolic dysfunction of the organs, insulin resistance (IR) and metabolic diseases (9, 10). Recently, evidence from a large number of observational studies has demonstrated that VAT is not only a major risk factor for NAFLD in obese individuals but may also be a key driving factor for NAFLD in lean individuals (11, 12). Furthermore, VAT has been shown to play a significant role in the progression of hepatic steatosis, inflammation, and fibrosis in NAFLD patients (13, 14). Therefore, accurate measurement of VAT is of crucial importance for the diagnosis/prediction and treatment assessment of NAFLD. Unfortunately, the high economic and technical costs of Magnetic Resonance Imaging technology, the gold standard measure of VAT, have made its clinical application for VAT measurement more limited (3, 15). Moreover, although various VAT surrogate markers based on simple anthropometric measurements have been developed, such as waist circumference (WC), body mass index (BMI), waist-to-height ratio (WHtR), waist-to-hip ratio, and visceral adiposity index (VAI), these surrogate indicators only provide rough estimates of VAT content from the perspective of body fat distribution and do not reflect the significant metabolic impact of VAT (16, 17).

Recently, Bello-Chavolla et al. developed a novel visceral adiposity score, known as METS-VF, and validated its significant superiority over other traditional obesity indicators in estimating VAT (18). The METS-VF score, which includes BMI, WHtR, fasting plasma glucose (FPG), high-density lipoprotein cholesterol (HDL-C), triglycerides (TG), age, and sex, provides a comprehensive estimation of the content and metabolic impact of VAT not only in terms of the content and distribution of body fat and glycolipid metabolism, but also takes into account age and sex differences in VAT. Currently, several studies have revealed the superiority of METS-VF over traditional obesity indicators in assessing and predicting the risk of metabolic diseases such as diabetes, hypertension, hyperuricemia, and chronic kidney dysfunction (19–23). However, the association between METS-VF and NAFLD and the potential value of METS-VF in identifying individuals at high odds of NAFLD have not been reported yet. Thus, the current study, based on a large sample population of the NAGALA cohort, aimed to explore the value of the application of the METS-VF in the identification of NAFLD and further assessed the important impact of age factors on the odds of NAFLD associated with the METS-VF and on the risk-identification ability of the METS-VF.

## Methods

### Data source and study population

To explore the relationship between the newly developed METS-VF and NAFLD, the current study performed a secondary analysis of data for subjects from the NAGALA dataset. The NAGALA dataset has been described in detail in a previous study by Okamura T et al. and uploaded to the Dryad public database for open sharing (https://doi.org/10.5061/dryad.8q0p192) (24). Briefly, the NAGALA cohort study is based on a health examination program called “human dock,” initiated in 1994 at Murakami Memorial Hospital. The program is designed to promote public health by continuously recruiting people for health check-ups at the hospital, with a focus on diabetes mellitus and NAFLD, as well as their risk factors, for follow-up and investigation. According to the purpose of the current study, we extracted data from 20,944 subjects who entered the NAGALA cohort between 1994 and 2016 and further set the following subject exclusion criteria: (i) excessive alcohol consumption (≥210 g/week for males or ≥140 g/week for females) (25); (ii) FPG at baseline more than 6.1 mmol/L; (iii) diabetes diagnosed at baseline; (iv) incomplete examination data; (v) patients with liver disease other than fatty liver; (vi) receiving any medication at baseline; (vii) unexplained withdrawal from the study.

### Ethics review

In the previous study, Professor Okamura T stated that the Murakami Memorial Hospital Ethics Committee had approved the NAGALA project and that each subject had signed a written informed consent for the use of data (26). As the current study was a secondary analysis of the NAGALA cohort study and all study procedures were in accordance with the Declaration of Helsinki and STROBE guidelines (S1 text), the Ethics Committee of Jiangxi Provincial People’s Hospital approved the current study and waived the procedure for obtaining written informed consent (IRB2021-066).

### Data collection and definition

Subjects’ demographic data as well as information on disease history and lifestyle habits (habits of smoking, drinking, and exercise) were collected by standard self-administered questionnaires. Height, WC, weight and blood pressure [diastolic blood pressure (DBP), systolic blood pressure (SBP)] were measured using standard methods by professional medical examiners in a quiet clinic room. After subjects fasting for at least 8 hours, their venous blood samples were collected by medical personnel and then analyzed using a laboratory automatic biochemical analyzer to measure the following biochemical indicators: FPG, TG, γ-glutamyltransferase (GGT), aspartate aminotransferase (AST), alanine aminotransferase (ALT), HDL-C, glycated hemoglobin (HbA1c), and total cholesterol (TC). Subsequently, the Hepatic Steatosis Index (HSI) values were calculated using the information of subjects’ ALT, AST, BMI, sex, and diabetic status; additionally, the Fatty Liver Index (FLI) values were determined by using TG, BMI, GGT, and WC values. Smoking habits were categorized as non, former, and current smoking. Drinking habits were classified based on the weekly alcohol intake of subjects as non/small (< 40 g/week), light (40-139 g/week), or moderate (140-209 g/week) drinking (25). Having a habit of exercise was defined as engaging in sports activities at least once a week (27).

### Calculation formula of VAT surrogate markers and indicators of hepatic steatosis


$$\begin{array}{l} \text{METS}-\text{VF}=4.466\;+\;0.011\;*\;\left[{\left(\text{Ln }\left(\left(\text{Ln }\left(2\;*\text{ FPG}+\text{TG}\right)*\text{ BMI}\right)/\left(\text{Ln }\left(\text{HDL}-\text{C}\right)\right)\right)\right)}^{3}\right]\\ +3.239\;*\;\left[{\left(\text{Ln}\left(\text{WHtR}\right)\right)}^{3}\right]+0.319\;*\text{ sex}+0.594\;*\;\left[\text{Ln }\left(\text{age}\right)\right] \end{array}$$

Note: Sex in the METS-VF formula is a binary response variable (male=1, female=0) (18).


VAI(male)=[WC/(39.68 + 1.88 * BMI)]*(TG/1.03)*(1.31/HDL−C)

(28)


VAI(female)=[WC/(36.58 + 1.89 * BMI)]*(TG/0.81)*(1.52/HDL−C)

(28)


$$\text{BMI}=\text{Weight}\left(\text{kg}\right)/{\left[\text{Height}\left(\text{m}\right)\right]}^{2}$$

WHtR=WC (cm)/Height (cm)

HSI=8*(ALT/AST)+BMI(+2, if females or diagnosed diabetes)

(29)


$$\begin{array}{l} \text{FLI}=100\;*\text{ EXP}\left(0.953\;*\text{ Ln}\left(\text{TG}\right)+0.139\;*\text{ BMI}+0.718\;*\text{ Ln}\left(\text{GGT}\right)+0.053\;*\text{ WC}-15.745\right)/\\ \left[1+\text{ EXP}\left(0.953\;*\text{ Ln}\left(\text{TG}\right)+0.139\;*\text{ BMI}+0.718\;*\text{ Ln}\left(\text{GGT}\right)+0.053\;*\text{ WC}-15.745\right)\right] \end{array}$$

(30)

### Diagnostic criteria for NAFLD

As mentioned earlier, NAFLD was diagnosed based on abdominal ultrasound results. Firstly, the ultrasound images of the subjects were collected by the ultrasonographer; subsequently, gastroenterology experts scored the following ultrasound image features and made the final diagnosis, including hepatorenal echo contrast (0-4 points), deep attenuation (0-2 points), liver brightness (0-4 points), and vascular blurring (0-2 points) (31). Additionally, we defined the subjects’ fatty liver status based on their HSI and FLI values, respectively. Those with HSI > 36 were classified as NAFLD, otherwise as Non-NAFLD; while those with FLI ≥ 60 were classified as NAFLD, otherwise as Non-NAFLD.

### Statistical analysis

We categorized the subjects of both sexes into two groups, respectively, according to whether or not they were diagnosed with NAFLD on imaging and described and compared the baseline characteristics between the two groups. The type of distribution of all indicators was determined using QQ plots, with normally distributed continuous data expressed as mean (standard deviation) and comparisons between groups using t-tests, while non-normally distributed data expressed as median (interquartile range) and comparisons between groups using the Mann-Whitney U test. Categorical data were presented as frequency (%), and group comparisons were performed using the chi-square test.

Stepwise-adjusted multivariate logistic regression models were built to explore the independent association between METS-VF and NAFLD for both sexes. Prior to this, we calculated variance inflation factor (VIF) values for all variables using multiple linear regression analyses and only adjusted for non-collinear variables with VIF < 5 in subsequent models (32). The continuous variable METS-VF was first included in Model 1 with initial adjustments for anthropometric parameters and lifestyle habit indicators (age, height, BMI, drinking habits, exercise and smoking); Model 2 further adjusted for laboratory measures (FPG, TC, HDL-C and HbA1c) and blood pressure (DBP) based on Model 1; and, finally, all noncollinear variables were adjusted in Model 3. Furthermore, we transformed METS-VF into a categorical variable using quintile function and included it in the above regression models, followed by a linear trend test. It is worth noting that considering the significant impact of age-related reproductive status and metabolic condition on visceral fat content and NAFLD risk (33, 34), we employed the OpenGL technique to fit surface elevation plots of the association between METS-VF, age, and NAFLD, based on the adjustment strategy from logistic regression Model 3, and displayed this joint relationship through 3D fitted surface plots (35, 36). Moreover, to further validate the utility of METS-VF in assessing fatty liver risk, we also investigated its correlation with other widely recognized hepatic steatosis assessment indices, HSI and FLI. We defined NAFLD separately using the cutoff values of HSI and FLI as the outcome variables and explored the independent association between METS-VF and the odds of NAFLD in the three established multivariate logistic regression models mentioned above.

We also plotted the ROC curves of METS-VF, WC, BMI, WHtR and VAI for identification of NAFLD in both sexes and calculated AUC values, diagnostic thresholds, sensitivities, specificities and Youden index of the corresponding indices; and subsequently compared the differences in diagnostic efficacies between the METS-VF and other VAT surrogate markers using the Delong test (37). Similarly, considering the important influence of the age, we further age-grouped subjects of both sexes in the ROC analyses based on the associations of age and METS-VF with NAFLD shown in the 3D fitted surface plots to explore the changes in the diagnostic efficacy of METS-VF for NAFLD at different ages in both sexes. We conducted all the above analyses using statistical software R language version 4.2.1, Empower(R) version 2.20, and Free Statistics version 1.7, and set the significance level at two-sided *P* < 0.05.

## Results

### Subject inclusion and baseline characteristics comparison

**Figure 1:**
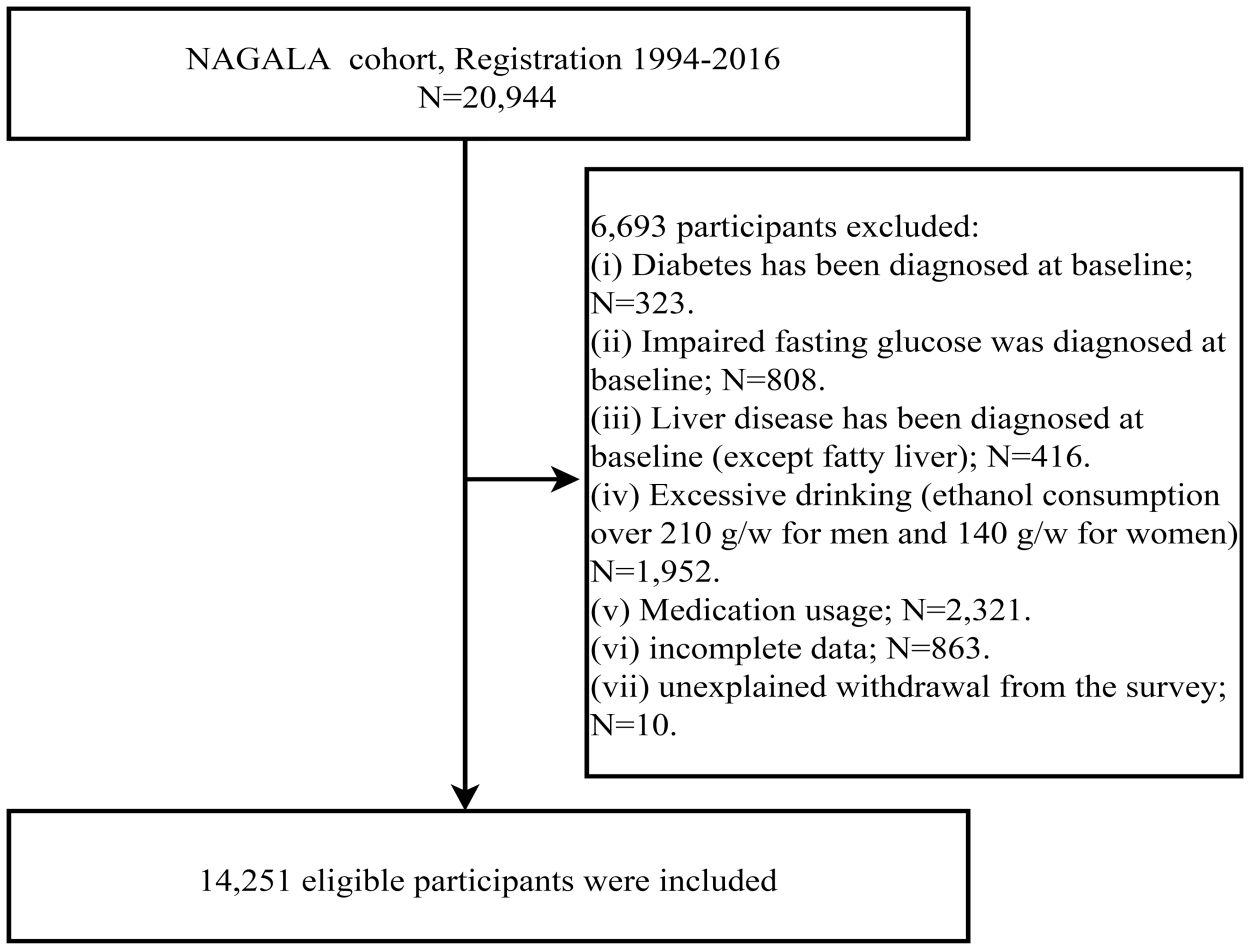
Flowchart of the selection process of study subjects.

After initially including 20,944 subjects, we excluded 323 individuals diagnosed with diabetes and 416 with liver disease (other than fatty liver), 808 with impaired fasting glucose, 1,952 with excessive alcohol consumption, and 2,321 receiving medication treatment. Additionally, 863 individuals with incomplete data and 10 with unknown reasons for study withdrawal were excluded; the detailed inclusion and exclusion process was shown in **Figure 1**. Ultimately, 14,251 subjects were identified, of whom 6,840 (48%) were females and 7,411 (52%) were males, with a mean age of 43.53 years. The baseline parameters of subjects in the non-NAFLD group and NAFLD group of both sexes were described and compared in **Table 1**. Firstly, we observed significant disparities in the prevalence of NAFLD between the two sexes, with a rate of 6.99% in females and 27.38% in males, nearly a four-fold difference. As for other baseline characteristics, similar trends were observed between the non-NAFLD and NAFLD groups for both sexes. NAFLD patients tended to be older, especially among female subjects, and had higher levels of body weight and abdominal fat content (weight, WC, WHtR, BMI, METS-VF), blood glucose (FPG, HbA1c), blood lipids (TC, TG), blood pressure (SBP, DBP), and liver function-related parameters (ALT, GGT, AST), but lower HDL-C levels and alcohol consumption. Additionally, height differed only in females, while exercise habits differed only in males, with female NAFLD patients having significantly shorter height and male NAFLD patients exhibiting significantly lower levels of physical activity.

**Table 1:** Baseline demographic, lifestyle, and laboratory characteristics of NAFLD and non-NAFLD subjects in both sexes.

Characteristic	Females			Males		
	Non-NAFLD	NAFLD	*P*-value	Non-NAFLD	NAFLD	*P*-value
No. of subjects	6362 (93.01%)	478 (6.99%)		5382 (72.62%)	2029 (27.38%)	
Age, year	42.0 (36.0-49.0)	49.0 (41.0-54.0)	<0.001	42.0 (36.0-50.0)	43.0 (38.0-50.0)	0.002
Height, cm	158.4 (5.4)	157.0 (5.3)	<0.001	170.9 (6.0)	170.6 (5.9)	0.084
Weight, kg	51.9 (7.1)	63.2 (10.0)	<0.001	64.6 (8.3)	74.3 (10.6)	<0.001
METS-VF	5.3 (0.7)	6.4 (0.5)	<0.001	5.8 (0.6)	6.5 (0.4)	<0.001
BMI, kg/m^2^	20.7 (2.6)	25.6 (3.6)	<0.001	22.1 (2.4)	25.5 (3.0)	<0.001
WC, cm	70.8 (7.3)	83.3 (8.9)	<0.001	78.0 (6.8)	86.6 (7.4)	<0.001
WHtR	0.4 (0.4-0.5)	0.5 (0.5-0.6)	<0.001	0.5 (0.4-0.5)	0.5 (0.5-0.5)	<0.001
SBP, mmHg	108.4 (13.8)	120.7 (16.0)	<0.001	116.0 (13.2)	124.0 (14.5)	<0.001
DBP, mmHg	67.0 (9.5)	75.1 (10.2)	<0.001	72.9 (9.3)	78.4 (10.1)	<0.001
TC, mmol/L	5.1 (0.9)	5.6 (0.9)	<0.001	5.1 (0.8)	5.4 (0.9)	<0.001
TG, mmol/L	0.5 (0.4-0.8)	1.0 (0.7-1.4)	<0.001	0.8 (0.6-1.2)	1.3 (0.9-1.9)	<0.001
HDL-C, mmol/L	1.6 (1.4-1.9)	1.3 (1.2-1.6)	<0.001	1.3 (1.1-1.5)	1.1 (1.0-1.3)	<0.001
FPG mmol/L	5.0 (0.4)	5.3 (0.4)	<0.001	5.3 (0.4)	5.4 (0.3)	<0.001
HbA1c, %	5.2 (0.3)	5.4 (0.3)	<0.001	5.1 (0.3)	5.3 (0.3)	<0.001
ALT, U/L	13.0 (11.0-17.0)	19.0 (15.0-26.0)	<0.001	18.0 (14.0-23.0)	29.0 (22.0-41.0)	<0.001
AST, U/L	16.0 (13.0-19.0)	18.0 (15.0-22.0)	<0.001	17.0 (14.0-21.0)	21.0 (17.0-26.0)	<0.001
GGT, U/L	12.0 (9.0-14.0)	15.0 (12.0-20.0)	<0.001	17.0 (14.0-24.0)	24.0 (18.0-35.0)	<0.001
Exercise habits, n (%)			0.335			<0.001
Non	5351 (84.1%)	410 (85.8%)		4300 (79.9%)	1720 (84.8%)	
Yes	1011 (15.9%)	68 (14.2%)		1082 (20.1%)	309 (15.2%)	
Drinking habits, n (%)			0.004			<0.001
Non/small	5986 (94.1%)	465 (97.3%)		3731 (69.3%)	1623 (80.0%)	
Light	376 (5.9%)	13 (2.7%)		1096 (20.4%)	273 (13.5%)	
Moderate				555 (10.3%)	133 (6.6%)	
Smoking habits, n (%)			0.664			0.067
None	5609 (88.2%)	427 (89.3%)		1952 (36.3%)	758 (37.4%)	
Past	382 (6.0%)	24 (5.0%)		1538 (28.6%)	615 (30.3%)	
Current	371 (5.8%)	27 (5.6%)		1892 (35.2%)	656 (32.3%)	

Values were expressed as mean (standard deviation) or medians (quartile interval) or n (%).

METS-VF, Metabolic Score for Visceral Fat; BMI, body mass index; WC, waist circumference; WHtR, waist to height ratio; TC, total cholesterol; SBP, systolic blood pressure; DBP, diastolic blood pressure; HbA1c, glycosylated hemoglobin; FPG, fasting plasma glucose; TG, triglyceride; HDL-C, high-density lipoprotein cholesterol; ALT, alanine aminotransferase; AST, aspartate aminotransferase; GGT, gamma-glutamyl transferase.

### Association of METS-VF with NAFLD

In the multicollinearity screening before conducting the multivariable logistic regression analysis, we excluded the collinear variables weight, TG, SBP, WC, and WHtR based on their calculated VIF values (**Supplementary Table 1**). **Table 2** presents the results of the multivariate logistic regression analyses. We found that from Model 1 to Model 3, the positive association between METS-VF and NAFLD was consistently maintained, although the strength of the association was progressively weaker in both sexes. In the fully adjusted Model 3, each unit increase in METS-VF was associated with a 3.33-fold and 3.12-fold increase in the odds of NAFLD for males and females, respectively; and after incorporating quintiles of METS-VF into the regression model, we found that compared to the reference level [first quintile (Q1)] of METS-VF, the OR values for NAFLD at Q2-Q5 levels were 5.47, 11.81, 21.09, and 23.72 in females, and 2.85, 5.67, 9.48, and 12.88 in males, respectively. Clearly, as METS-VF was increasing from Q1 to Q5, the odds of NAFLD appeared to have a larger increment in females, and the results of further trend analysis indicated that there was a linear positive correlation between METS-VF and the odds of NAFLD in both sexes. It’s worth noting that the correlation analysis with hepatic steatosis indices revealed a significant positive correlation between METS-VF and both HSI and FLI, with a stronger correlation observed with FLI (see **Supplementary Table 2**). Consistent results were obtained in the multivariate logistic regression analyses for NAFLD defined by HSI and FLI, all demonstrating a significant positive trend in the association between METS-VF and the odds of NAFLD, with METS-VF emerging as an independent risk factor for NAFLD (see **Supplementary Tables 3, 4**).

**Table 2:** Multivariable logistic regression analyses for the association between METS-VF and the incidence of NAFLD.

	OR (95%CI)		
	Model 1	Model 2	Model 3
Females			
METS-VF (continuous)	6.01 (4.04, 8.95)^*^	4.28 (2.84, 6.44)^*^	4.12 (2.73, 6.21)^*^
METS-VF Quintiles			
Quintile 1	Ref	Ref	Ref
Quintile 2	6.21 (1.86, 20.73)^*^	5.57 (1.67, 18.64)^*^	5.47 (1.63, 18.32)^*^
Quintile 3	15.97 (4.92, 51.81)^*^	12.34 (3.79, 40.24)^*^	11.81 (3.62, 38.58)^*^
Quintile 4	31.73 (9.68, 103.99)^*^	21.75 (6.58, 71.88)^*^	21.09 (6.37, 69.82)^*^
Quintile 5	43.91 (12.66, 152.34)^*^	26.52 (7.54, 93.28)^*^	23.72 (6.72, 83.76)^*^
*P* for trend	<0.0001	<0.0001	<0.0001
Males			
METS-VF (continuous)	9.53 (7.10, 12.80)^*^	5.88 (4.30, 8.04)^*^	4.33 (3.13, 5.99)^*^
METS-VF Quintiles			
Quintile 1	Ref	Ref	Ref
Quintile 2	3.61 (1.28, 10.23)^*^	3.10 (1.09, 8.79)^*^	2.85 (1.00, 8.13)
Quintile 3	9.74 (3.56, 26.61)^*^	6.96 (2.54, 19.08)^*^	5.67 (2.06, 15.63)^*^
Quintile 4	20.80 (7.57, 57.13)^*^	12.56 (4.55, 34.69)^*^	9.48 (3.41, 26.35)^*^
Quintile 5	36.95 (13.10, 104.19)^*^	18.90 (6.64, 53.78)^*^	12.88 (4.49, 37.01)^*^
*P* for trend	<0.0001	<0.0001	<0.0001

OR, odds ratio; CI, confidence interval; other abbreviations as in **Table 1**; *: P <0.05.

Model 1 was adjusted for age, height, BMI, exercise habits, smoking habits, and drinking habits.

Model 2 was adjusted for age, height, BMI, exercise habits, smoking habits, drinking habits, DBP, TC, HDL-C, FPG, and HbA1c.

Model 3 was adjusted for age, height, BMI, exercise habits, smoking habits, drinking habits, DBP, TC, HDL-C, FPG, and HbA1c, ALT, AST, and GGT.

**Figure 2:**
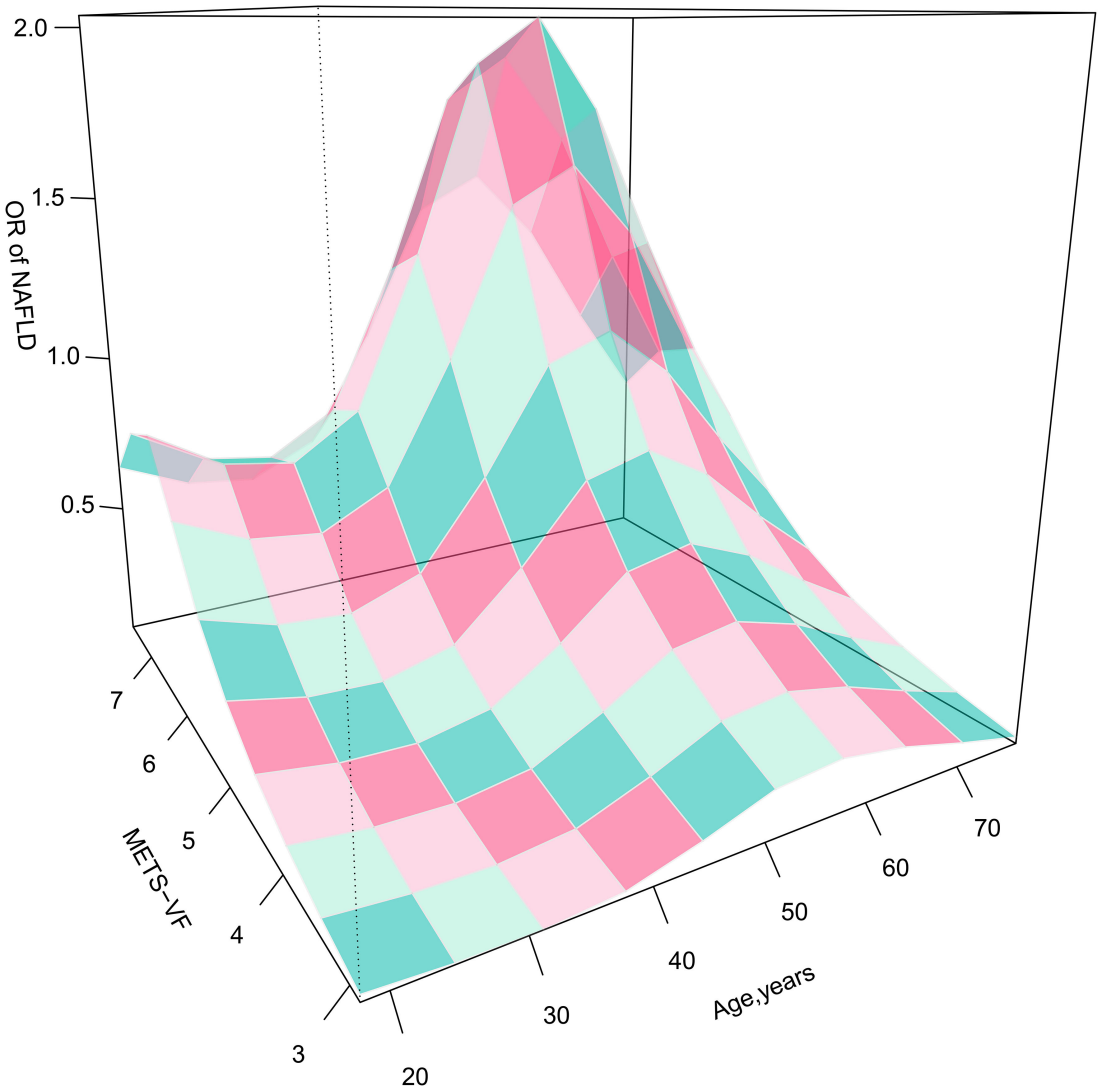
3D fitting surface plot of age, METS-VF and the odds of NAFLD in females. METS-VF, Metabolic Visceral Fat Score.

**Figure 3:**
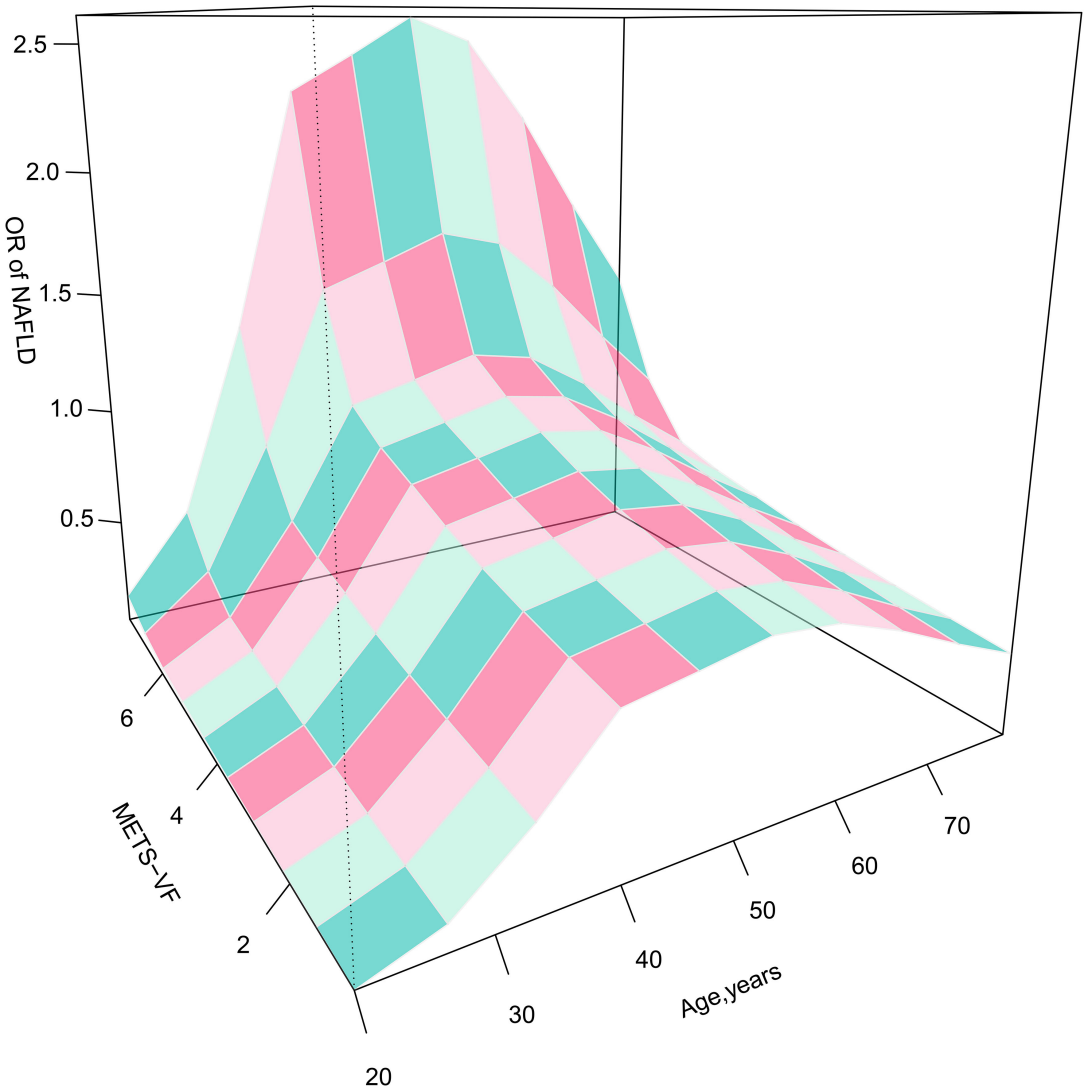
3D fitting surface plot of age, METS-VF and the odds of NAFLD in males. METS-VF, Metabolic Visceral Fat Score.

Furthermore, considering the strong association between age and VAT and the odds of NAFLD, we presented 3D fitted surface plots in **Figures 2**, **3** to illustrate the relationship between age and METS-VF with the odds of NAFLD in both sexes. We observed that with increasing METS-VF, the odds of NAFLD in females showed a clear linear upward trend, while this trend was weaker in males. Additionally, as age increased, females exhibited a slight decrease followed by a rapid increase in METS-VF-related odds of NAFLD. For females aged 18-40 years, aging did not seem to contribute to additional odds of NAFLD; however, in the 40-60 age group, aging significantly amplified the METS-VF-related odds of NAFLD, especially in individuals with higher METS-VF levels, leading to a greater combined effect of age and METS-VF on the odds of NAFLD. The METS-VF-related odds of NAFLD in females reached its peak at around 60 years of age and then gradually declined thereafter (**Figure 2**). In males, there was a linear increase in METS-VF-related odds of NAFLD between ages 20 and 45, followed by a plateau between ages 45 and 65 with a peak risk observed at around age 55, and subsequently, a gradual decline (**Figure 3**). It is noteworthy that although the above trends in the odds of NAFLD were consistent across all male subjects, there was a stronger combined effect of age and METS-VF on the odds of NAFLD when the male METS-VF value exceeded 6.2, leading to higher odds of NAFLD. Compared to male subjects with METS-VF values below 6.2, the same increase in age will result in significantly higher odds of NAFLD in male subjects with METS-VF values above 6.2, especially in the age group of 45 to 65 years, where the most significant changes in METS-VF-related odds of NAFLD occurred.

### Diagnostic value of METS-VF and other VAT surrogate markers for NAFLD

**Figure 4:**
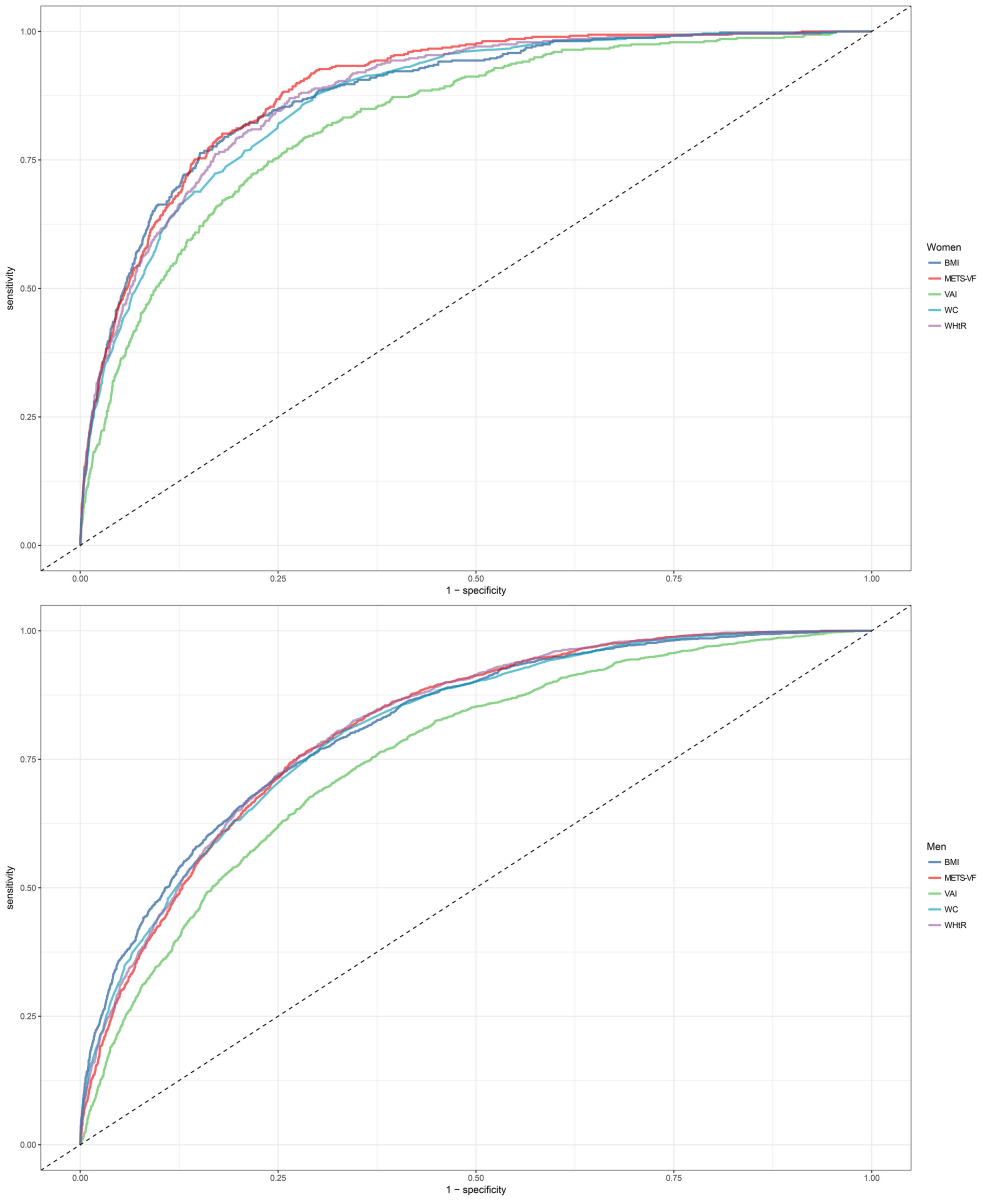
Receiver operating characteristic curves for predicting diabetes using BMI, WC, WHtR, VAI, METS-VF in males and females. METS-VF, Metabolic Score for Visceral Fat; BMI, body mass index; WC, waist circumference; WHtR, waist to height ratio; VAI, visceral adiposity Index.

**Table 3** shows the results of ROC analysis of VAT surrogate markers for identification of NAFLD. We found that METS-VF had very high diagnostic accuracy for NAFLD in females, with an AUC value of 0.8904 (95% CI: 0.8774-0.9034) and a diagnostic threshold of 5.8039, while the diagnostic value of METS-VF was relatively weaker for NAFLD in males, with an AUC value of 0.8123 (95% CI: 0.8021-0.8226) and a diagnostic threshold of 6.2287. After using the Delong test to compare the diagnostic performance of METS-VF with BMI, WC, VAI, and WHtR for NAFLD in both sexes, we found that METS-VF showed significantly superior diagnostic performance for NAFLD in females compared to other VAT surrogate markers (All Delong test *P <*0.05). However, for NAFLD in males, the diagnostic performance of METS-VF did not significantly differ from BMI, WC, and WHtR (Delong test *P >*0.05), but it was significantly better than VAI (**Figure 4**). In addition, considering the significant impact of age on the association between METS-VF and NAFLD, we further conducted stratified ROC analysis based on age groups. **Table 4** shows the results of age-stratified ROC analyses in both sexes. By comparing the AUC values, sensitivity, specificity and Youden index of METS-VF in different age groups of both sexes, we found that the diagnostic efficacy of METS-VF for NAFLD decreased with aging in both sexes, and that the AUC values of METS-VF in female subjects <45 years old, 45-60 years old and ≥60 years old were 0.9256, 0.8440 and 0.7778, whereas in male subjects, the AUC values for METS-VF were 0.8528, 0.7918 and 0.7686, respectively.

**Table 3:** Area under the ROC curve, Sensitivity, Specificity, Best threshold, and Youden index of METS-VF, BMI, WC, VAI, and WHtR to predict NAFLD.

	AUC	95%CI low	95%CI up	Best threshold	Specificity	Sensitivity	Youden index
Females							
METS-VF	0.8904	0.8774	0.9034	5.8039	0.7436	0.8828	0.6264
BMI^#^	0.8799	0.8648	0.8950	22.7565	0.8177	0.7950	0.6127
VAI^#^	0.8281	0.8098	0.8464	0.8228	0.7177	0.7929	0.5106
WHtR^#^	0.8790	0.8647	0.8932	0.4735	0.7356	0.8703	0.6059
WC^#^	0.8695	0.8545	0.8844	74.2500	0.7050	0.8745	0.5804
Males							
METS-VF	0.8123	0.8021	0.8226	6.2287	0.7231	0.7570	0.4801
BMI	0.8160	0.8055	0.8264	23.5555	0.7382	0.7309	0.4691
VAI^#^	0.7565	0.7445	0.7684	1.1117	0.7101	0.6770	0.3871
WHtR	0.8156	0.8054	0.8257	0.4736	0.6747	0.8053	0.4800
WC	0.8102	0.7998	0.8207	80.6500	0.6674	0.8034	0.4708

ROC, receiver-operating characteristic curve; AUC, area under the ROC curve; VAI, visceral adiposity Index; OR, Odds ratio; CI, confidence interval; Other abbreviations as in **Table 1**; #: P <0.05 compared with METS-VF.

**Table 4:** Area under the ROC curve, Sensitivity, Specificity, Best threshold, and Youden index of METS-VF to predict NAFLD in different age groups of both sexes.

	AUC	95%CI low	95%CI up	Best threshold	Specificity	Sensitivity	Youden index
Females							
<45	0.9256	0.9088	0.9425	5.7267	0.8178	0.9130	0.7308
45-60	0.8440	0.8213	0.8667	6.0652	0.7587	0.7774	0.5361
≥60	0.7778	0.6898	0.8657	6.0284	0.5220	0.9200	0.4420
Males							
<45	0.8528	0.8407	0.8648	6.0581	0.7452	0.7965	0.5417
45-60	0.7918	0.7742	0.8094	6.2412	0.5880	0.8591	0.4471
≥60	0.7686	0.7170	0.8202	6.6649	0.7245	0.6951	0.4196

ROC, receiver-operating characteristic curve; AUC, area under the ROC curve; Other abbreviations as in **Table 1**.

## Discussion

In this study involving 14,251 subjects, we found for the first time a significant positive association between METS-VF and the odds of NAFLD in both sexes, and this association was significantly affected by age in males and females. In males, there was a minimal combined effect of age and METS-VF when METS-VF values were less than 6.2; however, when male METS-VF values exceeded 6.2, the same increment in age resulted in significantly higher odds of NAFLD, corresponding to the elevated METS-VF levels. Furthermore, in female subjects aged 18-40 years, aging not only failed to increase the METS-VF-related odds of NAFLD, but instead, they experienced the lowest odds of NAFLD at around 32 years. However, in female subjects aged 40-60 years, age-related odds of NAFLD rapidly increased, particularly in individuals with higher METS-VF levels, resulting in more additional odds of NAFLD. In the ROC analysis, we also found that METS-VF showed significantly better diagnostic performance for NAFLD compared to other VAT surrogate markers, especially in the females; and further age-stratified ROC analysis revealed that METS-VF exhibited higher diagnostic value in younger age groups, particularly in female subjects younger than 45 years, where it achieved the highest diagnostic accuracy with an AUC value of 0.9256.

As previously mentioned, obesity is a recognized prominent physical characteristic of NAFLD patients (5), and in this study, both male and female NAFLD subjects exhibited significantly higher BMI values, all exceeding 25 kg/m^2^. However, with the deepening of research on NAFLD in recent years, an increasing number of individuals with normal BMI have been found to have NAFLD (6, 38, 39). A recent meta-analysis involving 105,786,383 subjects worldwide revealed that the prevalence of NAFLD varies from 25% to 50% across different countries; among these NAFLD patients, approximately 19.2% (95%CI: 15.9-23.0) were classified as lean individuals, while non-obese individuals accounted for a significant proportion of 40.8% (95%CI: 36.6-45.1) (39). Similarly, in a study by Liu CJ et al. on NAFLD in Asian populations, it was found that the proportion of non-obese NAFLD patients in Asia ranged from 17% in Taiwan to 75% in India (6). Evidence from these epidemiological studies has prompted numerous researchers to delve into the exploration of NAFLD risk factors beyond BMI, among which VAT is considered a more crucial independent risk factor, both in obese and non-obese NAFLD patients (11, 12, 40). There is already a wealth of evidence published regarding the role of VAT in the occurrence and progression of NAFLD; for instance, Chung et al. found an independent and dose-dependent relationship between VAT and elevated ALT levels in healthy individuals (41); and subsequently, in a histologically diagnostic-based study of NAFLD, Yu et al. confirmed that VAT was independently associated with non-alcoholic steatohepatitis and significant hepatic fibrosis (14, 42). In addition, VAT and subcutaneous adipose tissue (SAT) may have opposing effects on NAFLD, and the results of a large longitudinal cohort study with a median follow-up of 4.43 years by Kim D et al. suggested that VAT was independently associated with an increased odds of developing NAFLD whereas SAT was associated with regression of NAFLD (14). Evidence from the above observational studies suggested that accurate assessment and differentiation of VAT and SAT may be clinically important for risk assessment and prediction of NAFLD in the general population.

METS-VF is a new VAT estimator recently developed by Bello-Chavolla OY et al., and its detailed development and validation process have been described elsewhere (18). Briefly, Bello-Chavolla OY et al. first measured the VAT of subjects in the development cohort using dual-energy X-ray absorptiometry (DXA), and subsequently used subjects’ BMI, WHtR, FPG, HDL-C, TG, age, and sex as predictors, and curve-fitting yielded a VAT prediction model, METS-VF, that had the highest concordance with measurements from DXA. After obtaining the METS-VF, Bello-Chavolla OY et al. also measured the VAT of the subjects in the validation cohort using DXA, magnetic resonance imaging techniques, and bioelectrical impedance techniques, respectively, and validated and compared the accuracy of the METS-VF with other VAT surrogate markers for the estimation of VAT. The results showed that METS-VF had the strongest correlation with increased VAT in subjects in the validation cohort and had better predictive performance for increased VAT compared to BMI, VAI, LAP, WC and WHtR. Given the high accuracy and computational simplicity of METS-VF in predicting visceral obesity, more researchers have explored and confirmed its superior value in assessing and predicting the risk of visceral obesity-related diseases such as hyperuricemia, diabetes, CKD, and hypertension compared to other VAT surrogate indicators (19–23). However, to date, the relationship between METS-VF and NAFLD has not been reported. In this current study, we observed, for the first time in a large sample of individuals undergoing medical examinations, a significant and linear positive correlation between METS-VF and the odds of NAFLD in both sexes. Moreover, the results of ROC analysis demonstrated that METS-VF had significantly higher diagnostic value for NAFLD compared to other VAT surrogate markers, especially in females. These findings were consistent with previous research on METS-VF, all of which support that METS-VF was a superior diagnostic and predictive tool compared to traditional VAT surrogate indicators, with broad potential applications in diseases related to visceral obesity.

It is worth noting that considering the significant impact of age on the odds of NAFLD and VAT content in both sexes, investigating the isolated and combined effects of age and METS-VF on the odds of NAFLD may hold crucial implications for clinical prevention and risk stratification management of NAFLD in the general population (33, 34). The current study investigated the relationship between age and METS-VF with NAFLD based on established multivariate logistic regression models, and visually displayed the isolated and combined associations of both factors with the odds of NAFLD through 3D fitted surface plots (35, 36). In terms of the isolated effects of the two indicators on NAFLD, METS-VF was linearly and positively associated with the odds of NAFLD in both sexes, and an increase in METS-VF in females was associated with greater odds of NAFLD, which were consistent with the results of the logistic regression analyses [OR (Q5): females 23.72 > males 12.88]. In contrast, the influence of age on the odds of NAFLD showed distinct differences between the two sexes, with a U-shaped relationship observed in male age-related odds of NAFLD. In males, the odds of NAFLD increased gradually from young to middle age, but then decreased in elderly males; this pattern was highly consistent with the age-specific NAFLD prevalence curve for adult males proposed by Eguchi Y et al. (43). In females, the odds of NAFLD at the age of 18-40 years did not increase with age, and it is even still a low-risk stage around the age of 32 years; this may be related to the protective effect of estrogen in females of childbearing age, which encourages the deposition of adipose tissue more in the hips and thighs of females rather than in their visceral organs (44, 45). While, age-related odds of NAFLD elevated rapidly when females were aged between 40 and 60 years, possibly as a result of changes in hormonal levels and endocrine function resulting from declining ovarian function during menopause (46).

In terms of the combined effect of age and METS-VF on NAFLD, the odds of NAFLD were significantly affected in both sexes. In males, the magnitude of the combined effect of the two was mainly determined by the level of METS-VF. The combined effect of age and METS-VF was not significant when the METS-VF value in males was less than 6.2, whereas when the METS-VF value in males was greater than 6.2, the same increase in age will lead to higher odds of NAFLD in individuals with higher METS-VF levels. It is noteworthy that the optimal diagnostic threshold for METS-VF in males in the ROC analysis was 6.2287. Therefore, for males, VAT content may be a crucial factor contributing to the increased odds of NAFLD across all age groups (47, 48); considering the challenging aspects of aging-related adverse effects that are difficult to modify, maintaining METS-VF values below 6.2 may be a key strategy to reduce the odds of NAFLD in males. In females, age had a significantly stronger impact on METS-VF-related odds of NAFLD. In females aged 18-40 years, increasing age seemed to reduce the METS-VF-related odds of NAFLD; at the age of 40-60 years, the odds of NAFLD increased rapidly with both age and METS-VF levels, and their combined effect become more prominent; and at the age of 60 years, females had the highest odds of NAFLD at the same METS-VF level, a result that may be related to the unfavorable factors of altered perimenopausal reproductive status and endocrine metabolic disorders that occur with aging (48). On the other hand, females gradually lose the protective effect of estrogen against visceral fat accumulation during this stage, leading to an increased accumulation of VAT even with equivalent dietary fat intake compared to younger females, resulting in a further elevation of NAFLD risk (49). Therefore, for females aged 18-40, METS-VF is a more critical risk factor for NAFLD, and reducing visceral fat accumulation by lowering fat intake and increasing exercise may be the main way to reduce the odds of NAFLD for females at this stage; whereas, for females aged 40-60, not only should they focus on adopting a healthy lifestyle to reduce VAT accumulation, but also consider certain interventions such as estrogen replacement therapy to mitigate the adverse metabolic effects associated with aging and menopause (50). Additionally, we conducted age-stratified ROC analysis to explore the diagnostic value of METS-VF for NAFLD in different age groups among both sexes, which showed that METS-VF had higher diagnostic accuracy for the odds of NAFLD in the younger population, particularly in females under the age of 45, where the AUC value reached as high as 0.9256; indeed, the above findings were mutually corroborated by the 3D-fitted surface plot. Clearly, young females usually have a favorable metabolic status, and their odds of developing NAFLD don’t significantly increase with age; additionally, higher estrogen levels during this stage provide resistance against VAT accumulation. Therefore, METS-VF served as a sensitive indicator for monitoring NAFLD in young females, and when its level exceeded the optimal diagnostic threshold, there was greater confidence in diagnosing NAFLD.

### Research strengths and limitations

There are several strengths of the current study: (i) The present study has a large sample of medical examination population (n=14,251), on the basis of which we explored for the first time the risk assessment ability and diagnostic value of METS-VF for NAFLD through rational study design and statistically rigorous methodology, and therefore our results are relatively reliable. (ii) This study demonstrated that METS-VF had a higher diagnostic value than other VAT surrogate markers in the diagnosis of NAFLD, and fitted isolated and combined associations of age and METS-VF with the odds of NAFLD using 3D fitted surface plots. These findings based on the new methodology can better provide precise recommendations for NAFLD prevention in both sexes at different ages.

Limitations of the study: (i) The diagnosis of NAFLD in this study was based on the results of abdominal ultrasound rather than the gold-standard liver tissue biopsy, which may have led to the missed diagnosis of some patients with mild hepatic steatosis (51, 52). However, the use of invasive procedures in a large-scale health examination population is not in accordance with the Helsinki Declaration. (ii) The cross-sectional design of the current study prevented us from clarifying the causal relationship between METS-VF and the odds of NAFLD, which needs to be validated in further large longitudinal cohort studies. (iii) Although we have adjusted for a large number of confounders in the regression models, some residual confounding may still be present due to the fact that it is a secondary analysis of a previous study (53); furthermore, we were unable to update the dataset for the current study, which prevented us from further calculating liver fibrosis-related indices, such as the Fibrosis-4 index. It is necessary to explore the relationship between METS-VF and NAFLD and liver fibrosis in future research. (iv) The current study is a Japanese population-based study, and the applicability of the findings to other ethnic populations requires further validation.

## Conclusion

Our new findings suggested that METS-VF was a superior biomarker for diagnosing NAFLD compared to other VAT surrogate markers, especially for young females, where it had the highest diagnostic value. Additionally, considering the changes in the odds of NAFLD with age and METS-VF levels in both sexes, we recommended that males should strive to keep their METS-VF values below 6.2 to minimize the odds of NAFLD; for females, it was essential not only to maintain lower METS-VF levels but also to adopt specific interventions during the 40-60 age range to mitigate the adverse effects of aging and hormonal changes on the odds of NAFLD.

## Data availability statement

The original contributions presented in the study are included in the article/**Supplementary Material**. Further inquiries can be directed to the corresponding authors.

## Ethics statement

The studies involving humans were approved by the Ethics Committee of Jiangxi Provincial People’s Hospital. The studies were conducted in accordance with the local legislation and institutional requirements. The ethics committee/institutional review board waived the requirement of written informed consent for participation from the participants or the participants’ legal guardians/next of kin because As the current study was a secondary analysis of the NAGALA cohort study and all study procedures were in accordance with the Declaration of Helsinki and STROBE guidelines (S1 text), the Ethics Committee of Jiangxi Provincial People’s Hospital approved the current study and waived the procedure for obtaining written informed consent.

## Author contributions

MK: Data curation, Formal Analysis, Software, Validation, Writing – original draft. JQ: Formal Analysis, Software, Validation, Writing – original draft. DL: Formal Analysis, Software, Validation, Writing – original draft. CH: Formal Analysis, Validation, Writing – review & editing. SZ: Formal Analysis, Validation, Writing – review & editing. GS: Conceptualization, Project administration, Supervision, Writing – review & editing. YZ: Conceptualization, Data curation, Methodology, Project administration, Supervision, Writing – review & editing.
