# Supplementary material for: The newly proposed Metabolic Score for Visceral Fat is a reliable tool for identifying non-alcoholic fatty liver disease, requiring attention to age-specific effects in both sexes
Source: Front Endocrinol (Lausanne). 2023 Nov 27;14:1281524. doi: 10.3389/fendo.2023.1281524 (PMC10711077; doi:10.3389/fendo.2023.1281524)
Supplement: Supplementary file 1 [file Table_1.docx]

Supplementary Table 1: Collinearity diagnostics steps.

|  | Variance inflation factor | | | | | |
| --- | --- | --- | --- | --- | --- | --- |
|  | Step 1 | Step 2 | Step 3 | Step 4 | Step 5 | Step 6 |
| METS-VF | 17.4 | 17.4 | 17.3 | 5 | 5 | 5 |
| Age | 1.9 | 1.9 | 1.9 | 1.7 | 1.7 | 1.7 |
| Height | 88.7 | 49.6 | 1.9 | 1.5 | 1.4 | 1.4 |
| FPG | 1.5 | 1.5 | 1.5 | 1.5 | 1.5 | 1.4 |
| Weight | 426.4 | 168.5 | NA | NA | NA | NA |
| Smoking habits | 1.3 | 1.3 | 1.3 | 1.3 | 1.3 | 1.3 |
| Drinking habits | 1.2 | 1.2 | 1.2 | 1.2 | 1.2 | 1.2 |
| BMI | 228.9 | 98.7 | 5 | 4 | 4 | 4 |
| HDL-C | 2.3 | 2.3 | 2.3 | 2.1 | 2 | 2 |
| WHtR | 960.2 | 16.9 | 16.8 | NA | NA | NA |
| TG | 11.7 | 11.7 | 11.7 | 10.8 | NA | NA |
| TC | 1.5 | 1.5 | 1.5 | 1.5 | 1.4 | 1.4 |
| WC | 1208.9 | NA | NA | NA | NA | NA |
| Exercise habits | 1 | 1 | 1 | 1 | 1 | 1 |
| ALT | 4.1 | 4.1 | 4.1 | 4.1 | 4 | 4 |
| AST | 3.4 | 3.3 | 3.3 | 3.3 | 3.3 | 3.3 |
| GGT | 1.5 | 1.5 | 1.5 | 1.5 | 1.4 | 1.4 |
| HbA1c | 1.2 | 1.2 | 1.2 | 1.2 | 1.2 | 1.2 |
| SBP | 5.5 | 5.5 | 5.5 | 5.5 | 5.5 | NA |
| DBP | 5.6 | 5.6 | 5.6 | 5.5 | 5.5 | 1.4 |

Note-1: Variance inflation factor = 1/(1-R^2^). Abbreviations as in Table 1.

Note-2: The variables with variance inflation factor >5 will be regarded as collinear variables and cannot be included in the multiple regression model.

Supplementary Table 2: Spearman's rank correlation between METS-VF with FSI and FLI.

| Variable 1 | Variable 2 | Correlation β | | *P*-value |
| --- | --- | --- | --- | --- |
| METS-VF | HSI | 0.0922 | <0.001 | |
| METS-VF | FLI | 0.2720 | <0.001 | |

Abbreviations: HSI: Hepatitis Steatosis Index; FLI: Fatty Liver Index; other abbreviations as in Table 1.

Supplementary Table 3: Multivariable logistic regression analyses for the association between METS-VF and the incidence of NAFLD defined by HSI.

|  | OR (95%CI) | | |
| --- | --- | --- | --- |
|  | Model 1 | Model 2 | Model 3 |
| METS-VF (continuous) | 1.14 (1.07, 1.22)^*^ | 1.13 (1.04, 1.23)^*^ | 1.13 (1.04, 1.23)^*^ |
| METS-VF (quintiles) |  |  |  |
| Quintile 1 | Ref | Ref | Ref |
| Quintile 2 | 0.91 (0.77, 1.08) | 0.94 (0.78, 1.14) | 0.94 (0.78, 1.14) |
| Quintile 3 | 1.65 (1.41, 1.92)^*^ | 1.60 (1.34, 1.92)^*^ | 1.60 (1.33, 1.91)^*^ |
| Quintile 4 | 1.77 (1.51, 2.06)^*^ | 1.76 (1.47, 2.12)^*^ | 1.75 (1.46, 2.11)^*^ |
| Quintile 5 | 1.19 (1.02, 1.40)^*^ | 1.22 (0.99, 1.49) | 1.22 (1.00, 1.49) |
| *P* for trend | <0.0001 | <0.0001 | <0.0001 |

Abbreviations: OR: odds ratio; CI: confidence interval; other abbreviations as in Table 1; *: *P* <0.05.

Model 1 was adjusted for age, height, BMI, exercise habits, smoking habits, and drinking habits.

Model 2 was adjusted for age, height, BMI, exercise habits, smoking habits, drinking habits, DBP, TC, HDL-C, FPG, and HbA1c.

Model 3 was adjusted for age, height, BMI, exercise habits, smoking habits, drinking habits, DBP, TC, HDL-C, FPG, and HbA1c, ALT, AST, and GGT.

Supplementary Table 4: Multivariable logistic regression analyses for the association between METS-VF and the incidence of NAFLD defined by FLI.

|  | OR (95%CI) | | |
| --- | --- | --- | --- |
|  | Model 1 | Model 2 | Model 3 |
| METS-VF (continuous) | 1.33 (1.17, 1.52)^*^ | 1.28 (1.10, 1.49)^*^ | 1.27 (1.09, 1.49)^*^ |
| METS-VF Quintiles |  |  |  |
| Quintile 1 | Ref | Ref | Ref |
| Quintile 2 | 1.81 (1.19, 2.75)^*^ | 1.75 (1.15, 2.66)^*^ | 1.75 (1.15, 2.66)^*^ |
| Quintile 3 | 3.15 (2.15, 4.60)^*^ | 2.93 (1.98, 4.31)^*^ | 2.90 (1.97, 4.27)^*^ |
| Quintile 4 | 3.47 (2.39, 5.04)^*^ | 3.19 (2.15, 4.71)^*^ | 3.14 (2.12, 4.65)^*^ |
| Quintile 5 | 1.94 (1.30, 2.90)^*^ | 1.75 (1.13, 2.71)^*^ | 1.74 (1.12, 2.69)^*^ |
| *P* for trend | <0.0001 | <0.0001 | <0.0001 |

Abbreviations: OR: odds ratio; CI: confidence interval; other abbreviations as in Table 1; *: *P* <0.05.

Model 1 was adjusted for age, height, BMI, exercise habits, smoking habits, and drinking habits.

Model 2 was adjusted for age, height, BMI, exercise habits, smoking habits, drinking habits, DBP, TC, HDL-C, FPG, and HbA1c.

Model 3 was adjusted for age, height, BMI, exercise habits, smoking habits, drinking habits, DBP, TC, HDL-C, FPG, and HbA1c, ALT, AST, and GGT.
